# Diagnostic performance of Node Reporting and Data System (Node-RADS) for regional lymph node staging of gastric cancer by CT

**DOI:** 10.1007/s00330-023-10352-5

**Published:** 2023-10-24

**Authors:** Florian N. Loch, Katharina Beyer, Martin E. Kreis, Carsten Kamphues, Wael Rayya, Christian Schineis, Janosch Jahn, Moritz Tronser, Fabian H. J. Elsholtz, Bernd Hamm, Rolf Reiter

**Affiliations:** 1 Department of Surgery, Charité – Universitätsmedizin Berlin, corporate member of Freie Universität Berlin and Humboldt-Universität zu Berlin, Hindenburgdamm 30, 12203 Berlin, Germany; 2 Department of Surgery, Parkklinik Weißensee, Schönstraße 80, 13086 Berlin, Germany; 3 Department of Radiology, Charité – Universitätsmedizin Berlin, corporate member of Freie Universität Berlin and Humboldt-Universität zu Berlin, Hindenburgdamm 30, 12203 Berlin, Germany; 4 BIH Charité Digital Clinician Scientist Program, Berlin Institute of Health at Charité – Universitätsmedizin Berlin, BIH Biomedical Innovation Academy, Charitéplatz 1, 10117 Berlin, Germany

**Keywords:** Gastric cancer, Gastrectomy, Lymph nodes, Lymphadenectomy, Computed tomography

## Abstract

**Objectives:**

Diagnostic performance of imaging for regional lymph node assessment in gastric cancer is still limited, and there is a lack of consensus on radiological evaluation. At the same time, there is an increasing demand for structured reporting using Reporting and Data Systems (RADS) to standardize oncological imaging. We aimed at investigating the diagnostic performance of Node-RADS compared to the use of various individual criteria for assessing regional lymph nodes in gastric cancer using histopathology as reference.

**Methods:**

In this retrospective single-center study, consecutive 91 patients (median age, 66 years, range 33–91 years, 54 men) with CT scans and histologically proven gastric adenocarcinoma were assessed using Node-RADS assigning scores from 1 to 5 for the likelihood of regional lymph node metastases. Additionally, different Node-RADS criteria as well as subcategories of altered border contour (lobulated, spiculated, indistinct) were assessed individually. Sensitivity, specificity, and Youden’s index were calculated for Node-RADS scores, and all criteria investigated. Interreader agreement was calculated using Cohen’s kappa.

**Results:**

Among all criteria, best performance was found for Node-RADS scores ≥ 3 and ≥ 4 with a sensitivity/specificity/Youden’s index of 56.8%/90.7%/0.48 and 48.6%/98.1%/0.47, respectively, both with substantial interreader agreement (*κ* = 0.73 and 0.67, *p* < 0.01). Among individual criteria, the best performance was found for short-axis diameter of 10 mm with sensitivity/specificity/Youden’s index of 56.8%/87.0%/0.44 (*κ* = 0.65, *p* < 0.01).

**Conclusion:**

This study shows that structured reporting of combined size and configuration criteria of regional lymph nodes in gastric cancer slightly improves overall diagnostic performance compared to individual criteria including short-axis diameter alone. The results show an increase in specificity and unchanged sensitivity.

**Clinical relevance statement:**

The results of this study suggest that Node-RADS may be a suitable tool for structured reporting of regional lymph nodes in gastric cancer.

**Key Points:**

*• Assessment of lymph nodes in gastric cancer is still limited, and there is a lack of consensus on radiological evaluation.*

*• Node-RADS in gastric cancer improves overall diagnostic performance compared to individual criteria including short-axis diameter.*

*• Node-RADS may be a suitable tool for structured reporting of regional lymph nodes in gastric cancer.*

**Supplementary Information:**

The online version contains supplementary material available at 10.1007/s00330-023-10352-5.

## Introduction

Despite tremendous progress in the diagnosis and treatment of gastric cancer, prognosis is still poor with an overall 5-year survival rate of 31% [1, 2]. In this context, lymph node involvement of gastric cancer is of high prognostic importance for overall survival, as survival rates distinctly decrease with increasing numbers of metastatic lymph nodes [3]. Thus, regional lymph node involvement is a valid indication for perioperative chemotherapy [4]. Endoscopic ultrasound and computed tomography (CT) are the most established imaging modalities for regional nodal staging of gastric cancer [4, 5]. One major factor encumbering nodal staging is that malignant lymph nodes are not always enlarged and that benign lymph nodes may show reactive enlargement due to inflammation. Moreover, recommendations for optimal lymph node size thresholds vary widely from 6 to 12 mm [6–8]. Therefore, despite initial efforts and multimodal treatment planning, diagnostic accuracy and thus therapy planning are still limited, and there continues to be a lack of consensus in the radiological evaluation of lymph nodes.

In recent years, there has been an increasing demand for structured reporting using Reporting and Data Systems (RADS) to standardize the communication of oncological imaging findings, which is reflected by a widespread implementation of RADS for reporting the results of cancer imaging in various organs such as the breast (BI-RADS), prostate (PI-RADS), and liver (LI-RADS) [9–12]. Node-RADS 1.0 was introduced in 2021 for the structured assessment of lymph nodes in cancer and classifies the degree of suspicion of lymph node involvement using a synthesis of criteria [13]. In addition to the traditional “size” criterion, Node-RADS includes the criterion “configuration” with morphological categories of “texture,” “border,” and “shape” for the assessment of each individual lymph node. Those two overarching scoring criteria—size and configuration—are combined into the Node-RADS score ranging from 1 (very low) to 5 (very high likelihood of cancer).

We hypothesize that structured reporting of lymph node findings has the potential to improve regional nodal staging in gastric cancer. Therefore, we aimed at investigating the diagnostic performance of Node-RADS compared to the use of various individual criteria alone in assessing the regional lymph nodes on CT scans of gastric cancer patients using histopathology as reference.

## Material and Methods

### Patients

**Fig. 1:**
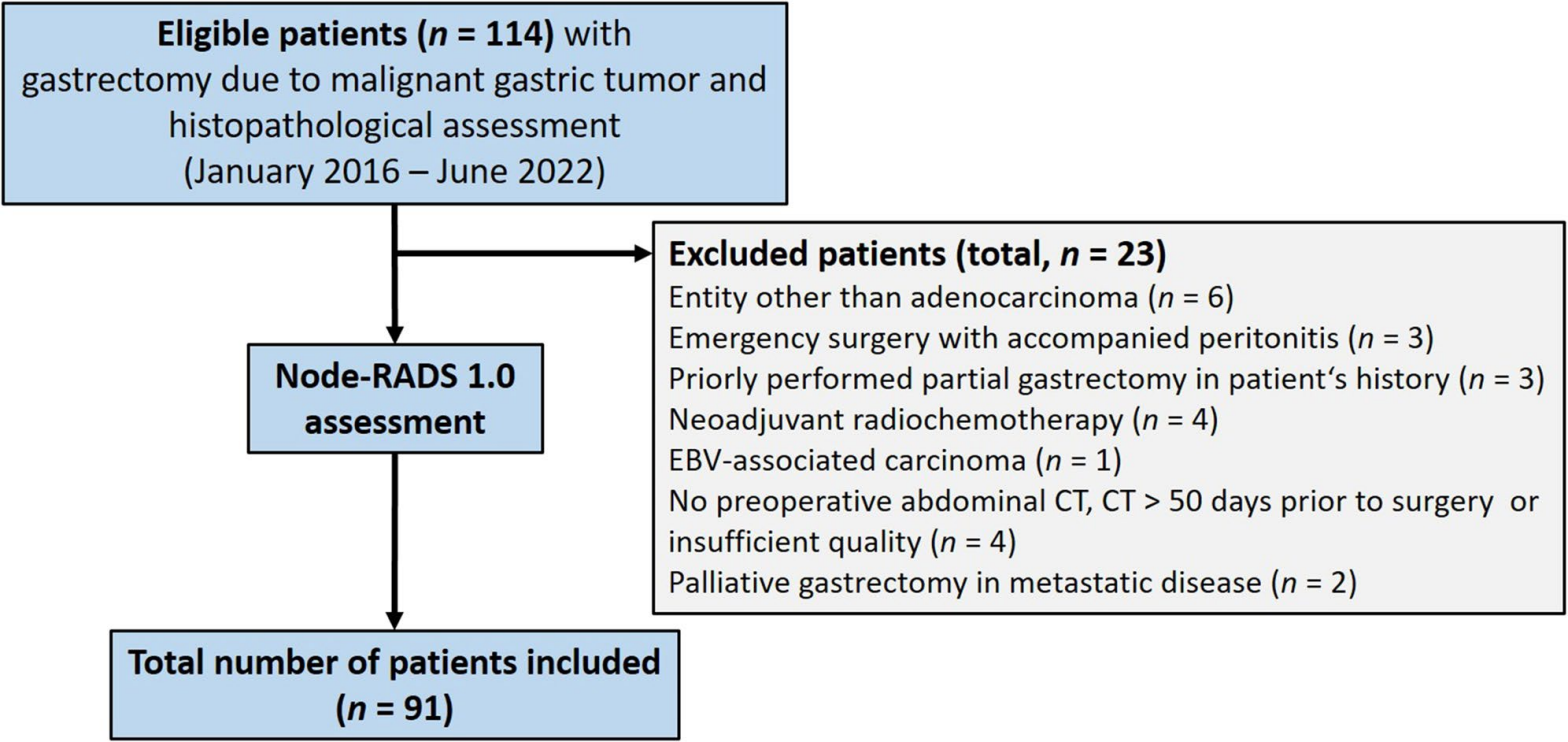
Flowchart of patient recruitment

The study was approved by the institutional review board of the Charité – Universitätsmedizin Berlin, and informed consent to participation was waived given the retrospective study design (No. EA4/114/18). In this retrospective single-center study, consecutive patients with histologically proven gastric adenocarcinoma who underwent surgery at the Department of Surgery, Campus Benjamin Franklin, Charité – Universitätsmedizin Berlin, Germany, between January 2016 and June 2022 were included. Inclusion and exclusion criteria are shown in Fig. 1.

### CT and Node-RADS

Radiological assessment was performed independently and blinded to histopathology by two readers (reader 1, R.R.: board-certified radiologist with over 10 years of experience in abdominal cross-sectional imaging; reader 2, J.J., radiology resident with 2 years of experience). Minimum quality was defined as contrast-enhanced CT with a slice thickness of at least 5 mm and all CT images were assessed for sufficient image quality by both radiologists [14, 15]. Lymph nodes were defined as regional according to the Japanese Gastric Cancer Association classification [16, 17]. Assessment was performed using Node-RADS 1.0 and assigning a score reflecting the likelihood of lymph node metastases as follows: 1 (very low), 2 (low), 3 (equivocal), 4 (high), 5 (very high) [13]. In brief, the Node-RADS score encompasses two overarching scoring criteria—“size” and “configuration”. For the size criterion, lymph nodes with a short axis ≥ 10 mm are considered “enlarged”, and lymph nodes with any axis ≥ 30 mm are considered “bulk”. This is the first step of the assessment and classifies the size in three categories: “normal”, “enlarged”, or “bulk”. The configuration criterion encompasses three categories with several corresponding features and scores: “texture” (“homogeneous”, 0 point; “heterogeneous”, 1 point; “focal necrosis”, 2 points; “gross necrosis”, 3 points), “border” (“smooth”, 0 point; “irregular/ill-defined”, 1 point), and “shape” (“any shape with preserved hilum”, 0 point; “kidney-bean-like or oval without fatty hilum”, 0 point; “spherical without fatty hilum”, 1 point). In the second step, a sum score of “configuration” (sum of points) is calculated ranging from 0 to 5 points which comprises the highest score of each configuration category. Finally, the sum score of “configuration” is weighted based on the size category to determine the Node-RADS score. For instance, a sum score of at least 3/2/1/0 results in Node-RADS 4/3/2/1 for “normal”-sized lymph nodes, and Node-RADS 5/4/3/2 for “enlarged” lymph nodes, respectively. All “bulk” lymph nodes are immediately classified as Node-RADS 5 without any additional criteria. For further details, including the three-level Node-RADS flowchart, please see Elsholtz et al [13]. Moreover, all criteria were assessed individually as were the three morphological subcategories of lymph nodes classified as having an irregular/ill-defined border (“lobulated”, “spiculated”, “indistinct”), as suggested in previous studies [18–20]. Finally, in addition to the existing criteria applied for Node-RADS, further analysis has been conducted to include the evaluation of “clustering of lymph nodes”, “hyperenhancement”, and “feeding vessel” as additional criteria. Interreader agreement of both radiologists was calculated.

### Surgery and perioperative treatment

All patients underwent oncologic total, subtotal, or transhiatal extended gastrectomy with D2/D2 + -lymphadenectomy of regional lymph nodes according to the guidelines of the Japanese Gastric Cancer Association [16, 17]. Patients underwent primary surgery or received prior preoperative chemotherapy. Patients with preoperative radiotherapy were excluded from this study.

### Histopathology

For the purpose of this study, the original histopathological reports on formalin-embedded surgical specimens were reviewed. Patients with any histologically proven regional lymph node metastases were classified as node-positive (pN +) and patients without any metastatic lymph nodes were classified as node-negative (pN-). Histopathological assessment of more than 16 lymph nodes was performed in each patient enrolled in the study. Tumors were classified according to their TNM stage using the 8th Edition of TNM Classification of Malignant Tumors [21].

### Comparison of CT and histopathology

Diagnostic performance was calculated separately for each individual criterion and for the Node-RADS score. CT examinations were considered node-positive (cN +) if at least one visible lymph node met the respective criterion used to identify metastatic involvement in the analysis. If no lymph node with the respective criterion was seen on CT, the examination was considered node-negative (cN-). These results (cN + /cN-) were used for calculation of sensitivity, specificity, and Youden’s index with histopathology as reference standard (pN + /pN-).

### Statistical analysis

Sensitivities and specificities were calculated for all criteria. As an index summarizing sensitivity and specificity, Youden’s index was calculated (sensitivity + specificity—1). The 95% confidence intervals (CI) were calculated for all values. Association between the number of visible lymph nodes on CT images and histopathological nodal involvement (pN +) was calculated using the Mann–Whitney *U* test. Area under the receiver operating characteristic curve (AUROC) analysis was performed for the numeric criterion size and Node-RADS based on the highest respective value per patient. Interreader agreement was calculated using Cohen’s kappa statistic (*κ*) and assessed as follows: *κ* < 0, no agreement; *κ* = 0.00–0.20, slight agreement; *κ* = 0.21–0.40, fair agreement; *κ* = 0.41–0.60, moderate agreement; *κ* = 0.61–0.80, substantial agreement; *κ* = 0.81–1.00, near perfect agreement. A *p* value of ≤ 0.05 was considered to indicate a statistically significant difference. Statistical analyses were performed using SPSS Statistics (Version 25.0., IBM Corp.) and MedCalc (Version 20.218, MedCalc Software Ltd).

## Results

Out of 114 patients initially screened for eligibility, 23 patients (20.2%) were excluded and 91 patients (79.8%) were included in this study (Fig. 1). Demographic and clinical characteristics of the 91 study patients are presented in Table 1. CT imaging has been performed within 1 week before surgery in 41.8% of cases, within 11 days (median) in 50.5% of cases, within 30 days in 93.4% of cases, and within 31–49 days in 6.6% of cases. Regional lymph nodes were visible on preoperative CT in 98.9% of all patients (patients: *n* = 90; lymph nodes: *n* = 443, median 4, range 0–15) and in 100% of patients with histopathological lymph node involvement (pN + , patients: *n* = 37; lymph nodes: *n* = 212, median 5, range 1–15). There was a significant association between the number of lymph nodes visible on preoperative CT and lymph node involvement (pN + , *p* = 0.01). Slice thickness of CT scans had the following distribution: 72 scans at 1 mm, 9 scans at 3 mm, 10 scans at 5 mm.

**Table 1:** Demographic and clinical characteristics of study patients and results of histopathological staging

Patients	*n* = 91
Age	
Median age (years)	66
Age range (years)	33–91
Gender	
Women	37 (40.7%)
Men	54 (59.3%)
Surgical procedure and perioperative therapy	
Total gastrectomy	44 (48.4%)
Subtotal gastrectomy	20 (22.0%)
Transhiatal extended gastrectomy	27 (29.7%)
Primary surgery	31 (34.1%)
Preoperative chemotherapy	60 (65.9%)
Time between CT and surgery	
Median	11 days
Range	1–49 days
Histopathological staging	
pN-	54 (59.3%)
pN +	37 (40.7%)
(y)pT1	19 (20.9%)
(y)pT2	9 (9.9%)
(y)pT3	38 (41.8%)
(y)pT4	17 (18.7)
ypT0	8 (8.8%)

### Size criterion

**Fig. 2:**
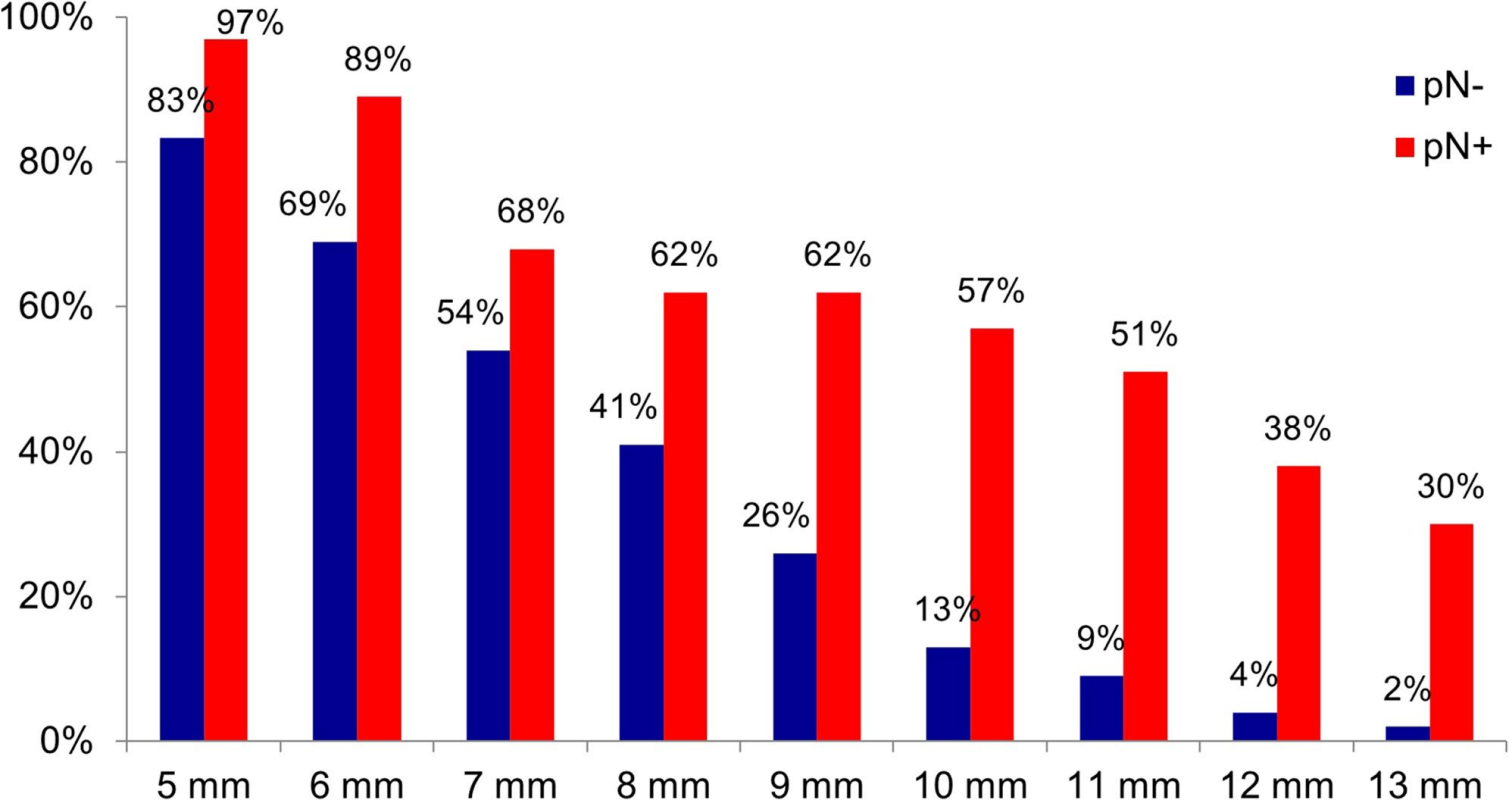
Size criterion. Percentage of patients with (pN + , red bars) and without (pN-, blue bars) histopathological lymph node involvement in whom a lymph node of respective short-axis diameter cut-off was visible on preoperative CT (5–13 mm). *n* = 443 lymph nodes in a total of 90 patients

Figure 2 shows the percentage of patients with (pN +) and without (pN-) histopathological lymph node involvement in whom a lymph node of the respective cut-off value in short-axis diameter (5–13 mm) was visible on preoperative CT. Table 2 presents sensitivity, specificity, and Youden’s index for each diameter cut-off from 5 to 16 mm. Lymph nodes with short-axis diameters of ≤ 9 mm were frequently seen in patients without histopathological lymph node involvement (pN-), giving rise to low specificity (< 75.0%). Lymph nodes with short-axis diameters ≥ 12 mm were rarely seen in either group of patients, giving rise to poor sensitivity (< 40.0%). The size criterion performed best with a short-axis diameter cut-off of 10 mm, resulting in 56.8% sensitivity, 87.0% specificity, and Youden’s index of 0.44. Additionally, the size criterion yielded an AUROC value of 0.72 (CI: 0.61–0.83, *p* < 0.01).

**Table 2:** Results for size criterion (short-axis diameter). Sensitivity, specificity, and Youden’s index for cut-offs from 5 to 16 mm. Cut-off with best diagnostic performance is shown in bold

Size criterion cut-off value	Sensitivity	Specificity	Youden’s Index
5 mm	97.3% CI: 85.8–99.9%	16.7% 7.9–29.3%	0.14 CI: 0.04–0.26
6 mm	89.2% CI: 74.6–97.0%	31.5% CI: 19.5–45.6%	0.21 CI: 0.05–0.35
7 mm	67.6% CI: 50.2–82.0%	46.3% CI: 32.6–60.4%	0.14 CI: 0.01–0.35
8 mm	62.2% CI: 44.8–77.5%	59.3% CI: 45.0–72.4%	0.21 CI: 0.02–0.41
9 mm	62.2% CI: 44.8–77.5%	74.1% CI: 60.3–85.0%	0.36 CI: 0.18–0.55
**10 mm**	**56.8%** **CI: 39.5–72.9%**	**87.0%** **CI: 75.1–94.6%**	**0.44** **CI: 0.24–0.60**
11 mm	51.4% CI: 34.4–68.1%	90.7% CI: 79.7–96.9%	0.42 CI: 0.23–0.58
12 mm	37.8% CI: 22.5–55.2%	96.3% CI: 87.3–99.5%	0.34 CI: 0.18–0.51
13 mm	29.7% CI: 15.9–47.0%	98.1% CI: 90.1–100%	0.28 CI: 0.14–0.43
14 mm	27.0% CI: 13.8–44.1%	98.1% CI: 90.1–100%	0.25 CI: 0.12–0.41
15 mm	16.2% CI: 6.2–32.0%	98.1% CI: 90.1–100%	0.14 CI: 0.04–0.31
16 mm	10.8% CI: 3.0–25.4%	100.0% CI: 93.4–100%	0.11 CI: 0.03–0.24

*CI*, 95% confidence interval

### Configuration criterion

**Fig. 3:**
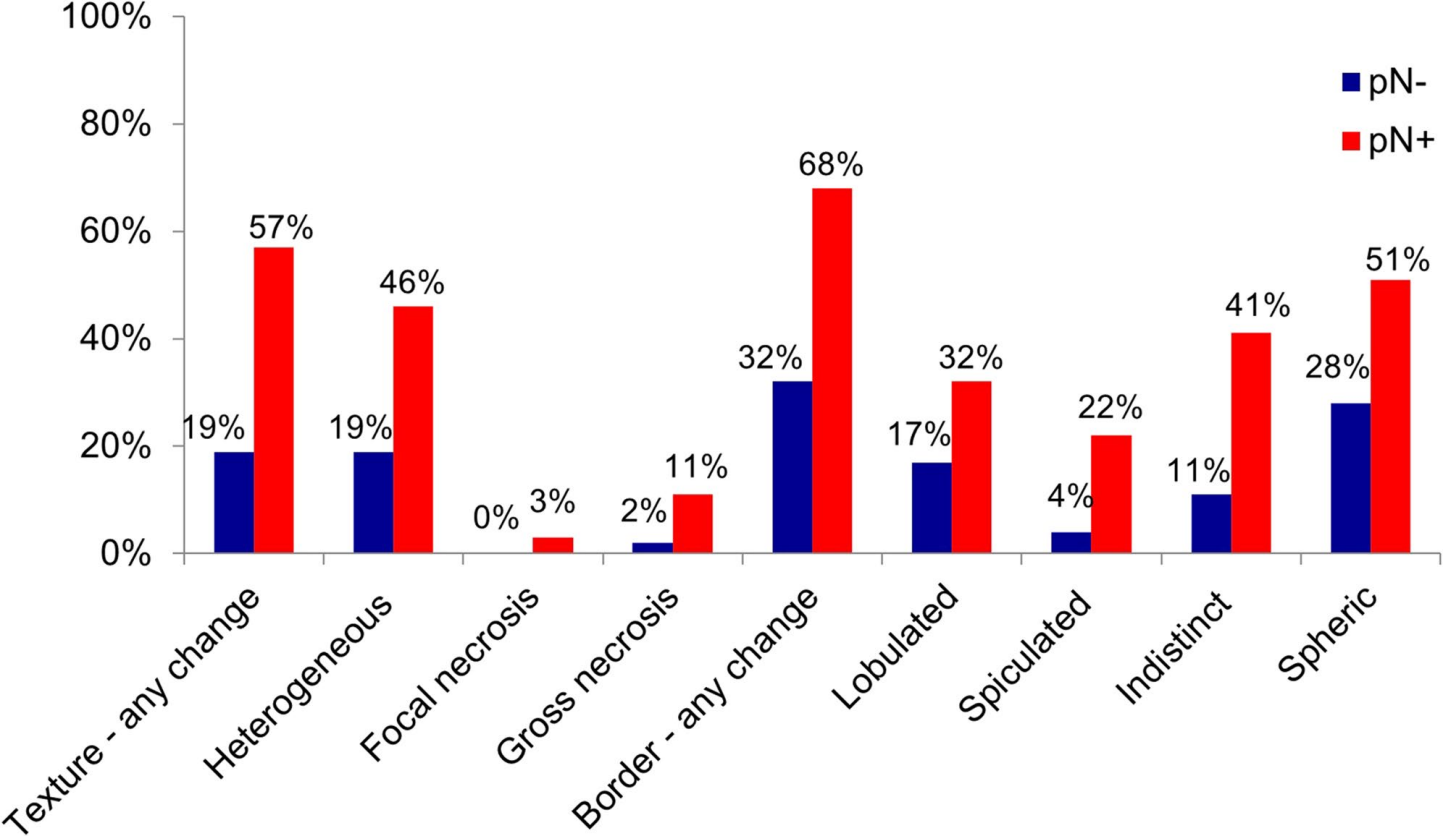
Configuration criterion. Percentage of patients with (pN + , red bars) and without (pN-, blue bars) histopathological lymph node involvement in whom a lymph node with the respective morphological criterion was visible on preoperative CT. *n* = 443 lymph nodes in a total of 90 patients

Figure 3 shows the percentage of patients with (pN +) and without (pN-) histopathological lymph node involvement in whom a lymph node was visible on preoperative CT according to its configuration. Table 3 compiles sensitivities, specificities, and Youden’s indices for these criteria. Three criteria—focal necrosis, gross necrosis, and spiculated border—were highly specific (> 95%) but rarely seen in either group of patients, resulting in sensitivities < 25% and Youden’s indices < 0.20. An “indistinct” or “heterogeneous” lymph node was seen in > 40% of patients with lymph node involvement (pN +) and specificity remained > 80% with resulting Youden’s indices of 0.29 and 0.27, respectively. Best performance for the configuration criterion was found for “any change in texture” with 56.8% sensitivity, 81.5% specificity, and Youden’s index of 0.38.

**Table 3:** Results for configuration criterion. Sensitivity, specificity, and Youden’s index for the respective morphological criterion. Feature with the best diagnostic performance is shown in bold

Configuration criterion	Sensitivity		Specificity	Youden’s Index
Texture—any change	**56.8%** **CI: 39.5–72.9%**		**81.5%** **CI: 68.6–90.7%**	**0.38** **CI: 0.18–0.57**
Heterogeneous	46.0% CI: 29.5–63.1%		81.5% CI: 68.6–90.7%	0.27 CI: 0.07–0.47
Focal necrosis	2.7% CI: 0.07–14.2%		100.0% CI: 93.4–100%	0.03 CI: 0.00–0.14
Gross necrosis	10.8% CI: 3.0–25.4%		98.1% CI: 90.1–100%	0.09 CI: 0.02–0.22
Border contour—any change	67.6% CI: 50.2–82.0%		68.5% CI: 54.4–80.5%	0.36 CI: 0.15–0.55
Lobulated border	32.4% CI: 18.0–49.8%		83.3% 70.7–92.1%	0.16 CI: 0.01–0.33
Spiculated border	21.6% CI: 9.8–38.2%		96.3% C: 87.3–99.5%	0.18 CI: 0.05–0.33
Indistinct border	40.5% CI: 24.8–57.9%		88.9% CI: 77.4–95.8%	0.29 CI: 0.12–48
Shape				
Spheric shape	51.4% CI: 34.4–68.1%		72.2% CI: 58.4–83.5%	0.24 CI: 0.04–0.44

*CI* 95% confidence interval

### Node-RADS

**Fig. 4:**
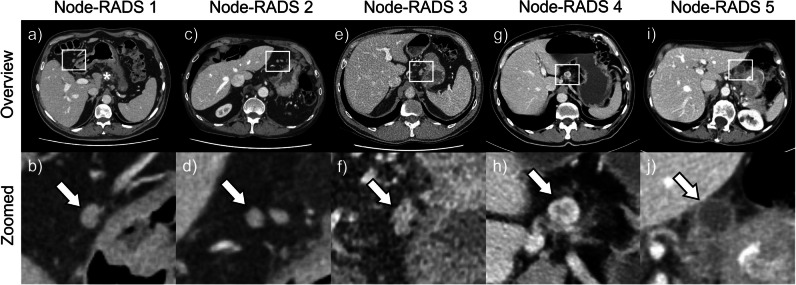
Representative examples of Node-RADS scores. White boxes indicate the zoomed area and arrows indicate the selected lymph node. **a**, **b** A 70-year-old man with node-positive T4a gastric cancer according to histopathology. The lymph node selected measures 11 × 8 mm and shows a homogeneous texture, smooth border, and kidney-bean-like shape. Asterisk indicates the pancreas. **c**, **d** A 78-year-old man with node-negative T3 gastric cancer. The lymph node measures 8 × 8 mm and shows a homogeneous texture, smooth border, and spherical shape. **e**, **f** A 60-year-old man with node-positive T3 gastric cancer. The lymph node measuring 17 × 11 mm is enlarged and shows a homogeneous texture, irregular (lobulated) border, and oval shape. **g**, **h** A 67-year-old man with node-positive T3 gastric cancer. The lymph node measuring 16 × 14 mm is enlarged and shows a heterogeneous texture, irregular (lobulated) border, and oval shape. **i**, **j** A 76-year-old woman with node-positive T4a gastric cancer. The lymph node measuring 18 × 18 mm is enlarged and shows gross necrosis and a spherical shape

**Fig. 5:**
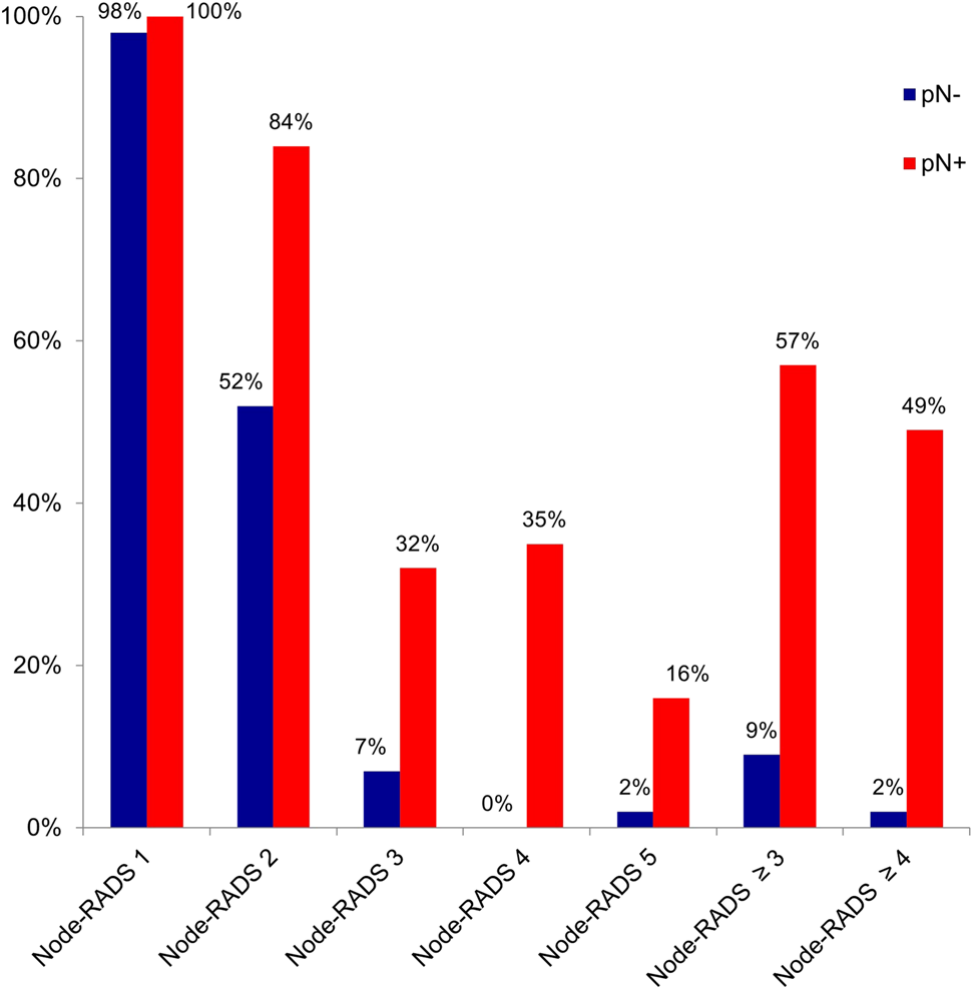
Node-RADS. Percentage of patients with (pN + , red bars) and without (pN-, blue bars) histopathological lymph node involvement in whom a lymph node with the respective Node-RADS score was visible on preoperative CT. *n* = 443 lymph nodes in a total of 90 patients

Figure 4 shows representative examples of the different Node-RADS scores. Distribution of Node-RADS scores in the study population was as follows (lymph node with the highest score on a per-patient basis): Node-RADS score 1, 34.1% (*n* = 31); Node-RADS score 2, 36.3% (*n* = 33); Node-RADS score 3, 7.7% (*n* = 7); Node-RADS score 4, 13.2% (*n* = 12); Node-RADS score 5, 7.7% (*n* = 7). As shown in Fig. 5, at least one lymph node with Node-RADS score 1 was seen in every patient with visible lymph nodes on preoperative CT. At least one lymph node with Node-RADS scores 3, 4, and 5 was seen in 32.4% (*n* = 12), 35.1% (*n* = 13), and 16.2% (*n* = 6) of patients *with* lymph node involvement (pN +), respectively. A lymph node with Node-RADS scores 3, 4, and 5 was seen in 7.4% (*n* = 4), 0% (*n* = 0), and 1.9% (*n* = 1) of patients *without* lymph node involvement (pN-), respectively. Table 4 presents sensitivities, specificities, and Youden’s indices for all five Node-RADS scores individually, for Node-RADS scores ≥ 3, and for Node-RADS scores ≥ 4. In 56.8% of patients with lymph node involvement (pN +), at least one lymph node with a Node-RADS score ≥ 3 was visible on preoperative CT. However, they were only seen in 9.3% of patients without lymph node involvement (pN-). The best performance for Node-RADS was found for scores ≥ 3 with 56.8% sensitivity, 90.7% specificity, and Youden’s index of 0.48 and for scores ≥ 4 with 48.6%, 98.1%, and 0.47, respectively. Moreover, Node-RADS scores ≥ 3 and ≥ 4 showed the best performance of all criteria investigated in this study. Additionally, Node-RADS yielded an AUROC value of 0.78 (CI: 0.67–0.88, *p* < 0.01).

**Table 4:** Results for Node-RADS. Sensitivity, specificity, and Youden’s index for combined and individual Node-RADS scores. Scores with the best diagnostic performance are shown in bold

Node-RADS	Sensitivity	Specificity	Youden’s Index
**Node-RADS score ≥ 3**	**56.8%** **CI: 39.5–72.9%**	**90.7%** **CI: 79.7–96.9%**	**0.48** **CI: 0.29–0.65**
**Node-RADS score ≥ 4**	**48.6%** **CI: 31.9–65.6%**	**98.1%** **CI: 90.1–100%**	**0.47** **CI: 0.30–0.63**
Node-RADS score 1	100.0% CI: 90.5–100%	1.9% CI: 0.05–9.9%	0.02 CI: 0.00–0.11
Node-RADS score 2	83.8% CI: 68.0–93.8%	48.1% CI: 34.3–62.2%	0.32 CI: 0.13–0.48
Node-RADS score 3	32.4% CI: 18.0–49.8%	92.6% CI: 82.1–97.9%	0.25 CI: 0.10–0.43
Node-RADS score 4	35.1% CI: 20.2–52.5%	100% CI: 93.4–100%	0.35 CI: 0.22–0.51
Node-RADS score 5	16.2% CI: 6.2–32.0%	98.1% CI: 90.1–100%	0.14 CI: 0.04–0.30

*CI* 95% confidence interval

### Additional criteria

Of all cases, “clustering” has been present in 17 cases (18.7%), “hyperenhancement” in two cases (2.2%) and “feeding vessel” in one case (1.1%). Sensitivities, specificities, and Youden’s indices for these criteria are presented in supplementary table S1. All three criteria were highly specific (> 90.0%). “Clustering” showed moderate to low sensitivity (32.4%), whereas “hyperenhancement” and “feeding vessel” showed low sensitivity (0.0–5.4%) due to their limited occurrence.

### Subgroup analysis of primary surgery versus neoadjuvant chemotherapy

Subgroup analysis for sensitivities, specificities, and Youden’s indices was performed for patients who received primary surgery versus patients who received neoadjuvant chemotherapy. Supplementary table S2 summarizes the results for both subgroups for a selection of criteria with the best diagnostic performance. In the subgroup of patients receiving neoadjuvant chemotherapy, Youden’s indices for all categories of criteria were consistently higher than in the subgroup receiving primary surgery: “size” (Youden’s index < 0.15 vs. > 0.40), “configuration” (Youden’s index < 0.10 vs. > 0.30), and Node-RADS (Youden’s index ≤ 0.25 vs. > 0.5).

### Interreader agreement

Interreader agreement results for a selection of criteria with the best diagnostic performance are listed in Table 5. For the size criterion, the best diagnostic performance was found for short-axis diameter cut-offs of 10 mm (Youden’s indexes of 0.44 and 0.36 for readers 1 and 2, respectively) and 9 mm (Youden’s indexes of 0.36 and 0.36, respectively)—both with substantial agreement (*κ* = 0.79 and 0.65, *p* < 0.01, respectively). For the configuration criterion, the best diagnostic performance was found for “texture – any change” (Youden’s indexes of 0.38 and 0.33 for readers 1 and 2, respectively) and “border contour – any change” (Youden’s indexes of 0.36 and 0.37, respectively)—both with moderate agreement (*κ* = 0.46 and 0.43, *p* < 0.01, respectively). For Node-RADS, the best diagnostic performance was found for Node-RADS scores ≥ 3 (Youden’s indexes of 0.48 and 0.52 for readers 1 and 2, respectively) and Node-RADS scores ≥ 4 (Youden’s indexes of 0.47 and 0.33 for readers 1 and 2, respectively)—both with substantial agreement (*κ* = 0.73 and 0.67, *p* < 0.01, respectively). For the additional criteria “clustering of lymph nodes”, “hyperenhancement”, and “feeding vessel”, interreader agreements were *κ* = 0.44, 1.00, and 0.66, respectively (*p* < 0.05).

**Table 5:** Interreader agreement (Cohen’s kappa, *κ*) of both readers for a selection of criteria and Node-RADS scores with the best diagnostic performance

	Sensitivity Reader 1	Specificity Reader 1	Youden’s Index Reader 1	Sensitivity Reader 2	Specificity Reader 2	Youden’s Index Reader 2	*κ*	*p* value
Size criterion Cut-off								
9 mm	62.2% CI: 44.8–77.5%	74.1% CI: 60.3–85.0%	0.36 CI: 0.18–0.55	56.8% CI: 39.5–72.9%	79.6% CI: 66.5–89.4%	0.36 CI: 0.16–0.55	0.79	*p* < 0.01
10 mm	56.8% CI: 39.5–72.9%	87.0% CI: 75.1–94.6%	0.44 CI: 0.24–0.60	48.6% CI: 31.9–65.6%	87.0% CI: 75.1–94.6%	0.36 CI: 0.17–0.53	0.65	*p* < 0.01
Configuration criterion								
Texture—any change	56.8% CI: 39.5–72.9%	81.5% CI: 68.6–90.7%	0.38 CI: 0.18–0.57	46.0% CI: 29.5–63.1%	87.0% CI: 75.1–94.6%	0.33 CI: 0.15–0.51	0.46	*p* < 0.01
Border contour—any change	67.6% CI: 50.2–82.0%	68.5% CI: 54.4–80.5%	0.36 CI: 0.15–0.55	51.4% CI: 34.4–68.1%	85.2% CI: 72.9–93.4%	0.37 CI: 0.18–0.56	0.43	*p* < 0.01
Spheric shape	51.4% CI: 34.4–68.1%	72.2% CI: 58.4–83.5%	0.24 CI: 0.04–0.44	43.2% CI: 27.1–60.5%	92.6% CI: 82.1–97.9%	0.36 CI: 0.19–0.52	0.23	*p* = 0.02
Node-RADS								
Node-RADS score ≥ 3	56.8% CI: 39.5–72–9%	90.7% CI: 79.7–96.9%	0.48 CI: 0.29–0.65	59.5% CI: 42.1–75.2%	92.6% CI: 82.1–97.9%	0.52 CI: 0.33–0.69	0.73	*p* < 0.01
Node-RADS score ≥ 4	48.6% CI: 31.9–65.6%	98.1% CI: 90.1–100%	0.47 CI: 0.30–0.63	35.1% CI: 20.2–52.5%	98.2% CI: 90.1–100%	0.33 CI: 0.19–0.50	0.67	*p* < 0.01

*CI* 95% confidence interval

## Discussion

In this study, we have investigated the diagnostic performance of Node-RADS for the structured reporting of regional lymph node status on CT scans obtained in patients with gastric cancer using histopathology as reference. Our results show that Node-RADS slightly improves overall diagnostic performance compared to the use of various individual criteria including short-axis diameter alone. Specifically, among all investigated criteria, the best performance was found for Node-RADS scores ≥ 3 and ≥ 4 with a sensitivity/specificity/Youden’s index of 56.8%/90.7%/0.48 and 48.6%/98.1%/0.47, respectively. Among individual criteria, the best performance was found for a short-axis diameter cut-off of 10 mm with sensitivity/specificity/Youden’s index of 56.8%/87.0%/0.44, respectively. We attribute the increased overall diagnostic performance of Node-RADS to the combined assessment of multiple features. While sensitivity remains unchanged, the higher specificity is probably based on the Node-RADS algorithm, which assumes a metastatic lymph node when more than one pathological feature is present. Such an increase in specificity is beneficial for most established RADS systems, such as those for liver or prostate cancer, where lesion characterization is more challenging than detection. Conversely, for gastric cancer, a higher sensitivity for identification of metastatic lymph nodes would be desirable. Nevertheless, Node-RADS slightly improved diagnostic performance in the assessment of regional lymph nodes in gastric cancer. The overall diagnostic performance for the subgroup receiving neoadjuvant chemotherapy has increased for all criteria. However, the number of patients with lymph node metastasis (pN +) in the subgroup receiving primary surgery was small with *n* = 8. Since this represents a low absolute number of cases, we have restrained from further conclusions at this point. Additionally, we have analyzed the diagnostic performance of criteria that could potentially be added to scoring systems such as the Node-RADS score. Lymph nodes with “hyperenhancement” or “feeding vessel” were seen in ≤ 2.5% of cases and are therefore of restricted use for a widely applicable scoring system. “Clustering”, however, was seen in 18.7% of cases (*n* = 17) with a noticeable tendency of presence in patients with nodal metastasis (pN +) yielding a sensitivity of 32.4% while remaining specific (90.7%). From a practical standpoint, the significance of peak diagnostic performance for Node-RADS scores of 3 and 4 lies in the ability to accurately identify and differentiate malignant lymph nodes from benign ones. While Node-RADS can provide valuable information, it should be noted that it is not a standalone solution to overcome the challenges of late cancer detection. However, it represents a step forward in providing a standardized approach to nodal staging. By refining and improving the diagnostic performance of Node-RADS, patient outcome could potentially be improved in the future by tailoring treatment planning. By improving nodal staging, unnecessary aggressive chemotherapy could be potentially avoided, minimizing side effects while ensuring appropriate treatment for patients. In cases of nodal metastasis (pN +), correct pretherapeutic nodal staging would ensure proper indication of neoadjuvant chemotherapy and, thus, improve the prognosis of patients [22]. Therefore, research aimed at enhancing the diagnostic performance of Node-RADS holds potential value for patients by addressing the need for more accurate diagnosis of gastric cancer. The substantial interreader agreement between the two radiologists (*κ* = 0.73 and 0.67 for Node-RADS ≥ 3 and ≥ 4, respectively) underlines the practical applicability of Node-RADS.

According to Elsholtz et al, the decision on how lymph nodes with Node-RADS scores of 3 should be reported depends on the stage and histological grade of the primary tumor [13]. In our study, categorical classification of regional Node-RADS-3 lymph nodes as metastatic (cN +) slightly improved the diagnostic performance of both reader 1 (Youden’s index = 0.48) and reader 2 (Youden’s index = 0.52, Table 5). Lucciola et al conducted a study using Node-RADS to evaluate pelvic lymph nodes in prostate cancer [23]. The authors found an increased specificity (1.00 vs. 0.08–0.19), while sensitivity decreased (0.17 vs. 0.96–1.00) and AUROC remained unchanged (0.58 vs. 0.57–0.60, respectively) compared to validated nomograms [23]. Leonardo et al investigated the performance of Node-RADS in bladder cancer and demonstrated a moderate-to-high overall accuracy for malignant lymph node invasion with an AUROC of up to 0.91, and the option of setting different cut-off values according to specific clinical scenarios [24]. Meyer et al assessed lung cancer patients using Node-RADS and radiomics [25]. They found that Node-RADS and several CT texture features have been associated with the malignancy of mediastinal lymph nodes, and concluded that both assessments could be translated in clinical routine in the future. In a meta-analysis of 10 studies investigating imaging modalities in gastric cancer, Kwee et al found that endoscopic ultrasonography, CT, MRI, and PET did not reliably confirm or rule out lymph node metastasis [26]. For preoperative CT, they found sensitivities of 62.5–91.9% and specificities of 50.0–87.9%. In another meta-analysis of 32 studies, conducted by Seevaratnam et al, an overall poor diagnostic performance with 77.2% sensitivity, 78.3% specificity, and Youden’s index of 0.56 was found for preoperative CT [27]. However, none of those studies assessing regional lymph nodes in gastric cancer has used structured reporting combing multiple morphological criteria. Besides structured reporting, artificial intelligence and radiomics could play a key role in the assessment of lymph node involvement in gastric cancer. In a large multicenter study, Dong et al have demonstrated a good discrimination of the number of lymph node metastases in locally advanced gastric cancer in different validation cohorts with an overall c-index of up to 0.82 (CI: 0.76–0.89) [28]. Moreover, they have shown that their nomogram had an increased performance compared to the standard clinical N stage and tumor size (*p* < 0.05). Their results suggest that a preoperative deep learning-based radiomics nomogram could provide baseline information for individual treatment planning of gastric cancer surgery.

Although encouraging, our study has limitations. First, the retrospective study design did not allow node-by-node comparison of CT and histopathology [29]. Nevertheless, we were able (i) to retrieve histopathological information on nodal staging from original histopathological reports in all patients included in this study, (ii) to reliably classify nodal status on a per-patient basis, and (iii) to include a relatively large number of more than 90 patients with 443 lymph nodes visible on preoperative CT. Future prospective studies involving close cooperation between radiology, surgery, and pathology hold the potential for node-by-node matching. Second, scans were assessed at different slice thicknesses ranging from 1 to 5 mm, which might have led to bias in size measurements. Nevertheless, all scans were primarily acquired in 1-mm slice thickness. This initial image acquisition at 1 mm was lost in some cases during data storage or transfer. Moreover, most scans (72 out of 91) have been assessed at 1-mm slice thickness. Third, we have investigated a small number of patients. Nevertheless, we have collected all cases matching strict inclusion and exclusion criteria to restrict confounding factors of lymph node alterations in a specialized center for gastric cancer ensuring contemporary therapeutic standards. Finally, since the introduction of Node-RADS in 2021, only three studies have been published to date that have investigated Node-RADS for lymph node staging in prostate cancer, bladder cancer, and lung cancer [23–25]. Therefore, experience is still limited, and diagnostic performance may improve with future versions. Further research and prospective multicenter studies are needed to validate our results.

In our study, we have investigated the diagnostic performance of Node-RADS for assessing regional lymph nodes in gastric cancer on CT scans using histopathology as reference. We show that the structured combination of size and configuration criteria for lymph node assessment slightly increases overall diagnostic performance compared to various individual criteria including short-axis diameter alone. Our results show that specificity increases while sensitivity remains unchanged. Node-RADS may be a suitable tool for structured reporting of regional lymph nodes in gastric cancer.

### Supplementary Information

Below is the link to the electronic supplementary material.

Supplementary file1 (PDF 111 KB)

## Abbreviations

AUROC: Area under the receiver operating characteristic curve
BI-RADS: Breast Imaging-Reporting and Data System
CI: Confidence intervals
cN-: Node-negative patients, based on imaging
cN+: Node-positive patients, based on imaging
CT: Computed tomography
LI-RADS: Liver Imaging-Reporting and Data System
PI-RADS: Prostate Imaging-Reporting and Data System
pN-: Node-negative patients, based on pathology
pN+: Node-positive patients, based on pathology
RADS: Reporting and Data System
κ: Cohen’s kappa statistic

## Declarations

### Guarantor

The scientific guarantor of this publication is Dr. Florian Nino Loch.

### Statistics and biometry

No complex statistical methods were necessary for this paper.

### Methodology

• retrospective

• diagnostic study

• performed at one institution
