# Supplementary material for: Diagnostic performance of Node Reporting and Data System (Node-RADS) for regional lymph node staging of gastric cancer by CT
Source: Eur Radiol. 2023 Oct 24;34(5):3183–93. doi: 10.1007/s00330-023-10352-5 (PMC11126430; doi:10.1007/s00330-023-10352-5)
Supplement: Supplementary file 1 — Supplementary file1 (PDF 111 KB) [file 330_2023_10352_MOESM1_ESM.pdf]

Diagnostic Performance of Node Reporting and Data System (Node-RADS) for Regional Lymph Node Staging of Gastric Cancer by CT

Electronic Supplementary Material (ESM)

Eur Radiol (2023) Loch FN, Beyer K, Kreis ME et al

Additional Criteria	Sensitivity	Specificity	Youden's Index
Clustering	32.4%<br>CI: 18.0%-49.8%	90.7%<br>CI: 79.7%-96.9%	0.23<br>CI: 0.08-0.42
Hyperenhancement	5.4%<br>CI: 0.66-18.2%	100.0%<br>CI: 93.4%-100.0%	0.05<br>CI: 0.00-0.16
Feeding Vessel	0.0%<br>CI: 0.0-9.5%	98.1%<br>CI: 90.1%-100.0%	-0.02<br>CI: -0.13-0

Supplementary Table S1. Results for additional criteria. Sensitivity, specificity and Youden's index for the additional criteria clustering, hyperenhancement and presence of feeding vessel. CI: 95% confidence interval.

	Primary surgery (*n* = 31)			Neoadjuvant chemotherapy (*n* = 60)		
	**Sensitivity**	**Specificity**	**Youden's Index**	**Sensitivity**	**Specificity**	**Youden's Index**
**Size criterion cut-off value**						
9 mm	25.0% CI: 3.2-65.1%	78.3% CI: 56.3-92.5%	0.03 CI: 0.00-0.10	72.4% CI: 52.8-87.3%	71.0% CI: 52.0-85.8%	0.43 CI: 0.17-0.63
10 mm	25.0% CI: 3.2-65.1%	87.0% CI: 66.4-97.2%	0.12 CI: 0.01-0.46	65.5% CI: 45.7-82.1%	87.1% CI: 70.2-96.4%	0.53 CI: 0.29-0.73
**Configuration criterion**						
Texture – any change	25.0% CI: 3.2-65.1%	82.6% CI: 61.2-95.0%	0.08 CI: 0.00-0.26	65.5% CI: 45.7-82.1%	80.6% CI: 62.5-92.5%	0.46 CI: 0.19-0.64
Border contour – any change	37.5% CI: 8.5-75.5%	69.6% CI: 47.1-86.8%	0.07 CI: 0.01-0.24	75.9% CI: 56.5-89.7%	67.7% CI: 48.6-83.3%	0.44 CI: 0.18-0.64
Spheric shape	25.0% CI: 3.2-65.1%	69.6% CI: 47.1-86.8%	-0.05 CI: -0.17-0.01	58.6% CI: 38.9-76.5%	74.2% CI: 55.4-88.1%	0.33 CI: 0.06-0.54
**Node-RADS**						
Node-RADS score ≥ 3	25.0% CI: 3.2-65.1%	91.3% CI: 72.0-98.9%	0.16 CI: 0.01-0.58	65.5% CI: 45.7-82.1%	90.3% CI: 74.2-98.0%	0.55 CI: 0.32-0.73
Node-RADS score ≥ 4	25.0% CI: 3.2-65.1%	100.0% CI: 85.2-100.0%	0.25 CI: 0.00-0.63	55.2% CI: 35.7-73.6%	96.8% CI: 83.3-99.9%	0.52 CI: 31.5-69.2

Supplementary Table S2. Comparison of subgroups with primary surgery vs. neoadjuvant chemotherapy for criteria with best diagnostic performance. CI: 95% confidence interval.
